# Predicting Future Incidences of Cardiac Arrhythmias Using Discrete Heartbeats from Normal Sinus Rhythm ECG Signals via Deep Learning Methods

**DOI:** 10.3390/diagnostics13172849

**Published:** 2023-09-03

**Authors:** Yehyun Kim, Myeonggyu Lee, Jaeung Yoon, Yeji Kim, Hyunseok Min, Hyungjoo Cho, Junbeom Park, Taeyoung Shin

**Affiliations:** 1 Synergy A.I. Co., Ltd., Seoul 07573, Republic of Korea; ykim102@synergyai.co (Y.K.); brstar96@synergyai.co (M.L.); jaeung@synergyai.co (J.Y.); 2 Department of Cardiology, Ewha Womans University Mokdong Hospital, Seoul 07985, Republic of Korea; lexie6169@gmail.com; 3 Tomocube Inc., Daejeon 34141, Republic of Korea; min6284@gmail.com (H.M.); phelahab@gmail.com (H.C.); 4 Department of Urology, Ewha Womans University Mokdong Hospital, Seoul 07985, Republic of Korea

**Keywords:** arrhythmia risk prediction, atrial fibrillation risk prediction, artificial intelligence

## Abstract

This study aims to compare the effectiveness of using discrete heartbeats versus an entire 12-lead electrocardiogram (ECG) as the input for predicting future occurrences of arrhythmia and atrial fibrillation using deep learning models. Experiments were conducted using two types of inputs: a combination of discrete heartbeats extracted from 12-lead ECG and an entire 12-lead ECG signal of 10 s. This study utilized 326,904 ECG signals from 134,447 patients and categorized them into three groups: true–normal sinus rhythm (T-NSR), atrial fibrillation–normal sinus rhythm (AF-NSR), and clinically important arrhythmia–normal sinus rhythm (CIA-NSR). The T-NSR group comprised patients with at least three normal rhythms in a year and no atrial fibrillation or arrhythmias history. Clinically important arrhythmia included atrial fibrillation, atrial flutter, atrial premature contraction, atrial tachycardia, ventricular premature contraction, ventricular tachycardia, right and left bundle branch block, and atrioventricular block over the second degree. The AF-NSR group included normal sinus rhythm paired with atrial fibrillation or atrial flutter within 14 days, and the CIA-NSR group comprised normal sinus rhythm paired with CIA occurring within 14 days. Three deep learning models, ResNet-18, LSTM, and Transformer-based models, were utilized to distinguish T-NSR from AF-NSR and T-NSR from CIA-NSR. The experiments demonstrated the potential of using discrete heartbeats in predicting future arrhythmia and atrial fibrillation incidences extracted from 12-lead electrocardiogram (ECG) signals alone, without any additional patient information. The analysis reveals that these discrete heartbeats contain subtle patterns that deep learning models can identify. Focusing on discrete heartbeats may lead to more timely and accurate diagnoses of these conditions, improving patient outcomes and enabling automated diagnosis using ECG signals as a biomarker.

## 1. Introduction

Cardiac arrhythmias, encompassing conditions such as atrial fibrillation, are among the leading causes of concern in cardiovascular health. The insidious nature of these conditions, often manifesting asymptomatically or with minimal symptoms, renders them particularly elusive to standard detection methods [1,2,3]. The stakes of such undetected irregularities are alarmingly high, with potential outcomes ranging from debilitating strokes to heart failures and, in the most severe instances, culminating in sudden cardiac death [4].

The gravity of atrial fibrillation, a prominent subtype of arrhythmias, lies in its strong correlation with intensified risks of both stroke and heart failure [5,6,7,8,9]. This association highlights the pressing need for effective early detection mechanisms and prompt interventions. Despite the profound clinical implications, these conditions often go undetected, only becoming evident when they result in more severe outcomes.

The motivation behind our study is to bridge this detection gap. Recognizing the challenges faced in identifying these conditions early, we delved into innovative methods aimed at enhancing the screening process. By refining the current diagnostic paradigms, we believe we can bring a robust solution that aids in the proactive management of atrial fibrillation and other arrhythmias [10,11]. Through this endeavor, our motivation is clear: to mitigate the potential complications and enhance the quality of life for patients across the globe.

Electrocardiogram (ECG) recording includes 1-dimensional time series data that measure the heart’s electrical activity, and it is a valuable tool for diagnosing and monitoring arrhythmias and atrial fibrillation. Recent studies have demonstrated the potential of deep learning techniques in predicting the future incidence of arrhythmias and atrial fibrillation using ECG signals [12,13,14,15,16]. Previous approaches to ECG analysis have mainly concentrated on using whole 12-lead EKG recordings as the input for deep learning models due to the popularity of two-dimensional CNNs in analyzing various data types, such as auditory signals that were transformed into two-dimensional image data. However, applying the same approach to ECG signals may not be optimal due to the challenges posed by the complex and noisy nature of the electrical signals generated by the heart, which are superimposed on various noise sources, such as muscle movement, respiration, and electrical interference from other equipment.

**Figure 1:**
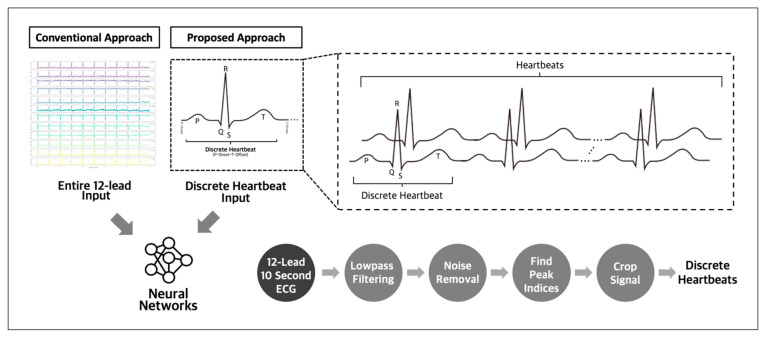
Conventional and proposed input approach for ECG analysis.

The utilization of discrete heartbeats as input data has been identified as a more optimal approach for detecting subtle abnormalities indicating future incidences of atrial fibrillation and other arrhythmias [17,18], compared to using whole 12-lead ECG recordings as the input. This approach enables the detection of critical temporal events, improving the performance of predictive models. Compared to using complete 12-lead ECG recordings as the input data, this approach allows for more focused analysis and reduces the need for larger datasets (Figure 1). Adopting this method facilitates the identification of the key indicators of potential cardiac issues, enhancing the accuracy of predictions.

To further optimize the approach for identifying subtle abnormalities in ECG signals, our methodology for predicting future cardiac events from normal sinus rhythm relies exclusively on the ECG signal, without using additional patient data, such as electronic medical records, that contain potentially sensitive or private information, such as age, gender, medical history, family history of heart disease, medication use, lifestyle factors (smoking and alcohol consumption), and comorbid conditions (hypertension or diabetes). While utilizing such data could enhance the accuracy of ECG prediction algorithms, deep learning models may focus more on medical records than ECG signals, leading to biased prediction results.

The primary aim of our study is to demonstrate that utilizing discrete heartbeats extracted from 10-s 12-lead sinus rhythm ECGs as inputs yields superior results compared to using an entire 12-lead heartbeat as the input for predicting future incidences of cardiac arrhythmias and atrial fibrillation. We conducted two distinct experiments: one for predicting the future incidence of atrial fibrillation, and another for predicting arrhythmias with prediction windows of 14 days. The reason for conducting separate experiments for arrhythmia and atrial fibrillation, despite atrial fibrillation being a type of arrhythmia, is to precisely analyze and understand the distinct characteristics and patterns associated with each condition. Isolating atrial fibrillation as a separate experiment allows for a more focused investigation into the unique features and predictive factors specific to it. The chosen prediction windows were aligned with the typical duration of wearing cardiac event monitors, which ranged up to 14 days. Evaluating the effectiveness of our approach in predicting clinically important arrhythmias within these windows provides insight into its potential usefulness in clinical practice. Moreover, our approach’s reliance on the ECG signal makes it a practical and feasible solution for clinical implementation, given that ECGs are routinely performed in clinical settings. Our study results indicate that using discrete heartbeats as the input yielded superior results compared to the conventional approach and could be a valuable tool for healthcare providers in predicting future cardiac arrhythmias from normal sinus rhythm and improving patient care and disease management.

## 2. Materials and Methods

### 2.1. Data Information and Study Population

We included 134,447 patients with 326,904 ECGs acquired from two Ewha Womans University Hospitals in Mokdong and Seoul, Republic of Korea, between May 2017 and May 2022. Raw ECGs were obtained from Philips (236,645 ECGs) and General Electric (90,259 ECGs) ECG machines in XML format. Philips ECGs are standard 10-s, 12-lead ECGs with a sampling rate of 500 Hz. GE ECGs are 10-s, 8- or 12-lead ECGs with a sampling rate of 500 Hz. The 8-lead ECGs from the General Electric ECG machine were reconstructed to 12 leads using Einthoven’s law and Goldberger’s equations [19].

### 2.2. Study Group Selection

We categorized 326,904 ECG datasets into three groups: true–normal sinus rhythm (“T-NSR”), atrial fibrillation–normal sinus rhythm (“AF-NSR”), and clinically important arrhythmia–normal sinus rhythm (“CIA-NSR”). We defined arrhythmias based on several criteria, which included atrial fibrillation, atrial flutter, atrial arrhythmia, premature ventricular contraction, right and left bundle branch block, and any atrioventricular block exceeding the second degree. Each of these conditions holds clinical significance and necessitates medical intervention.

#### 2.2.1. Study Group Selection with Automated Labels

**Figure 2:**
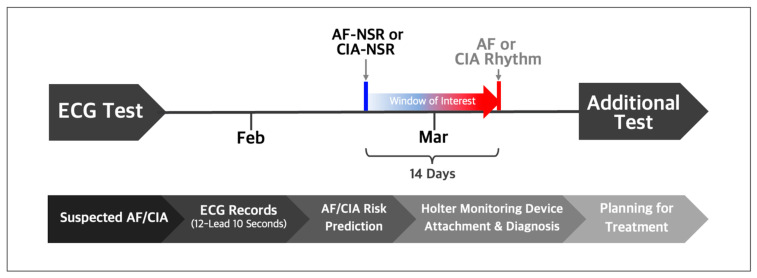
Normal sinus rhythm ECGs labeled by automatic symptom analysis reports were selected if AF or CIA occurred within 14 days after the respective normal sinus rhythm ECGs. Trained practitioners validated and relabeled the selected normal sinus rhythm as AF-NSR or CIA-NSR.

For the T-NSR group, we considered patients who recorded a minimum of three ECGs displaying normal sinus rhythm over 12 months and who had no documented history of atrial fibrillation or other arrhythmias. From this pool of T-NSR ECGs, we randomly selected one ECG per patient. The AF-NSR group comprised ECGs explicitly labeled as normal sinus rhythm and which had a corresponding ECG showing atrial fibrillation or atrial flutter within the subsequent 14 days. The CIA-NSR group included normal sinus rhythm paired with ECGs that showed CIA occurring within 14 days after the initial normal sinus rhythm reading, as illustrated in Figure 2.

To ensure the integrity of our dataset, we omitted any ECGs that were flawed or had missing or inconclusive interpretations from the T-NSR, AF-NSR, and CIA-NSR groups. To uphold consistency in our findings and focus on the adult demographic, we also excluded the ECG records of individuals younger than 18.

#### 2.2.2. Study Group Selection with Manual Labels

**Figure 3:**
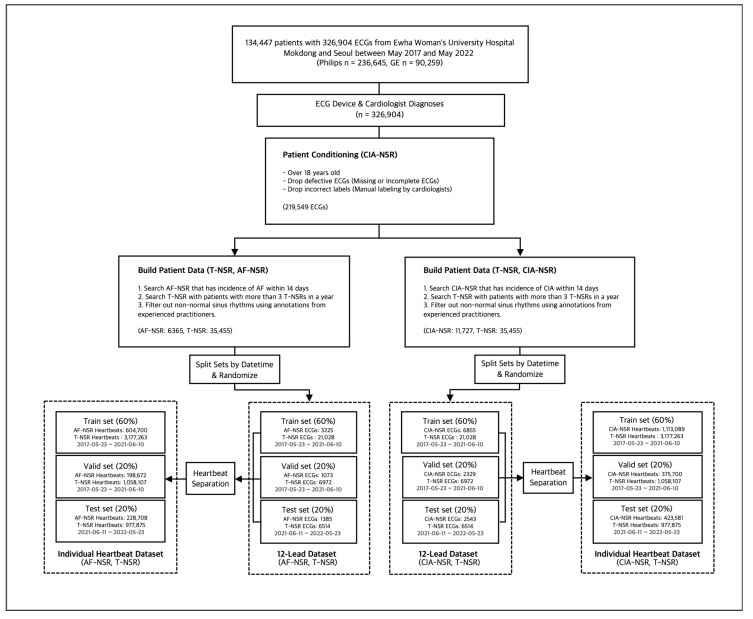
Study group selection procedure for T-NSR/AF-NSR and T-NSR/CIA-NSR.

Each ECG interpretation, thus far, was determined by automatic symptom analysis reports from the Philips and GE ECG machines. To ensure the accuracy of our data, we converted all selected ECGs into waveform images. We asked trained practitioners with more than five years of experience in cardiology to manually annotate them. Any ECGs with discrepancies between the automatic diagnosis and manual annotations were excluded from the study. To ensure robust model evaluation and simulate real-world scenarios, we partitioned the dataset based on the dates of the ECG scans and the patients who underwent them. The training and validation set spanned from 23 May 2017 to 10 June 2021, while the test set covered 11 June 2021 to 23 May 2022. For convenience in the learning context, train and validation set separation was performed on the ECG scan level. Following these selection processes, we obtained each group’s final number of ECGs, as shown in Figure 3.

### 2.3. Signal Data Preprocessing

**Figure 4:**
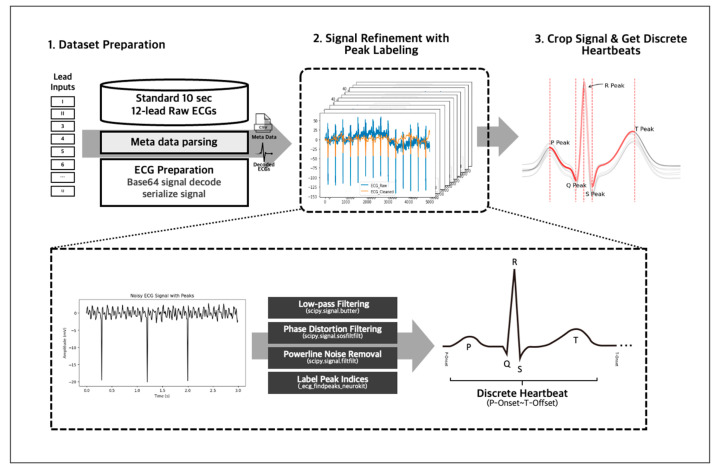
The 10-s 12-lead ECG signals were decoded from Base 64 and denoised using IIR Butterworth SOS and powerline noise filters. The clean signals were then segmented into individual heartbeats using a QRS peak detection algorithm via the NeuroKit2 library.

We employed several preprocessing techniques on our 10-s 12-lead ECG signal data to obtain accurate and reliable data (Figure 4). First, we decoded the data from Base 64 encryption and then passed it through an IIR Butterworth SOS and powerline noise filters with a moving average kernel for denoising and cleansing. Next, we segmented the denoised lead signals into individual heartbeats using a QRS peak detection algorithm, and this resulted in approximately 130 individual heartbeats for a 10-s 12-lead ECG, representing the PQRST complex per single ECG signal data. Any unrecognizable heartbeats were omitted from the data to ensure accuracy and consistency. The denoising, cleansing, and PQRST complex segmentation using peak detection algorithm were handled using the NeuroKit2 library [20], allowing for efficient and standardized data processing. After the individual data preprocessing, we inherited the 12-lead EKG’s annotation to the individual heartbeats to train with the individual heartbeats.

### 2.4. Overview of the Model Development

Table 1 and Table 2 shows ECGs and discrete heartbeat statistics used for training, validating, and testing the model. For the analysis of the one-dimensional discrete heartbeats and the whole 12-lead ECG signals, we employed popular deep learning architectures, which are ResNet-18, Conv1D with long short-term memory (LSTM), and Conv1D with transformer [21,22,23,24,25].

#### 2.4.1. Model Input Length

Given that every architecture integrates a convolutional layer as its initial layer, we standardized the length of individual heartbeats to match the mean length across all observed discrete heartbeats, set at 700. Heartbeats exceeding this length were truncated accordingly, whereas shorter ones were zero-padded. For the 12-lead ECG signal, we established a consistent signal length of 5000. We consciously abstained from utilizing interpolation techniques to resize the signals, as this could potentially introduce undue signal distortion.

#### 2.4.2. Model Architectures

**Figure 5:**
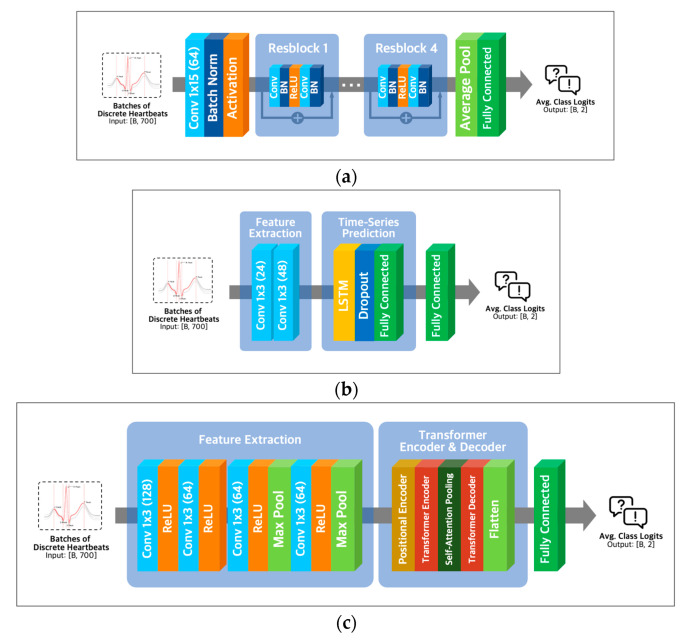
Model architectures. (**a**) ResNet-18 architecture; (**b**) LSTM architecture with Conv-1D layer; (**c**) transformer architecture with Conv-1D layer.

ResNet-18 extracts essential features of the input using convolution operations like various convolutional neural networks. To solve the vanishing gradient problem of CNN architectures [26], ResNet-18 utilizes residual learning with skip connection, as shown in Figure 5a.

Combining Conv1D and LSTM layers (Figure 5b) in a neural network architecture can capture local and long-range temporal patterns in sequential data. Conv1D layers are adept at detecting local patterns, while LSTM layers excel at modeling longer-term dependencies [27]. Alternatively, a combination of Conv1D and transformer layers (Figure 5c) can capture both local and global dependencies in the input data, with transformer layers being well-suited for modeling global dependencies [28] and Conv1D layers being effective for detecting local patterns.

#### 2.4.3. Model Parameters and Thresholds

**Figure 6:**
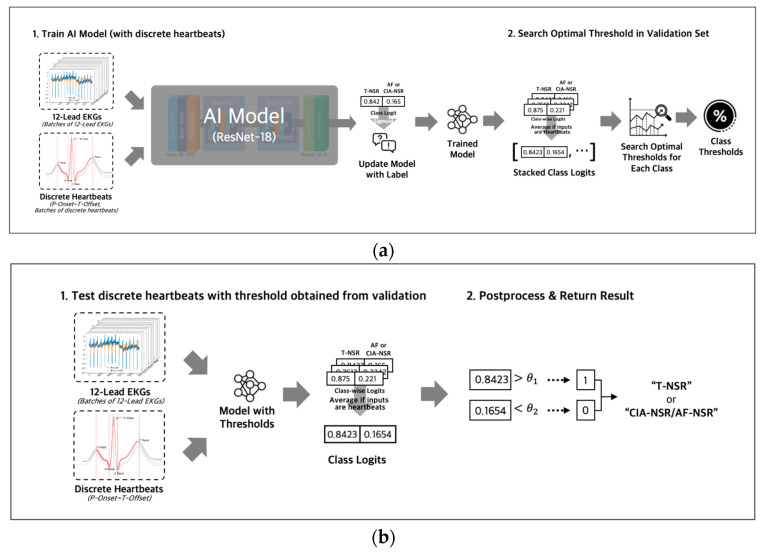
(**a**) Both inputs of entire 12-lead ECG and discrete heartbeats separated from 12-lead ECG were used to train AI Models with different architectures. All logit values from discrete heartbeats were averaged to represent the final logit value for the 12-lead ECG. Optimal thresholds were determined in the validation phase and then were saved along with the model weights; (**b**) test phase loaded model weights and threshold value that were saved in the training phase, then evaluated the ECG as T-NSR or CIA-NSR by comparing the threshold value and the final logit value.

During the training phase (Figure 6a), we used binary cross-entropy with logits loss and AdamW optimizer with an initial learning rate of 0.0001 to optimize the model’s parameters. The output of the fully connected layer was passed through a sigmoid function to obtain a probability value for each class, ranging from 0 to 1. For the discrete heartbeat input in the validation phase, we gathered the probability scores for discrete heartbeats that were separated from the same ECG; then we averaged all the probability scores of discrete heartbeats to represent the final probability score for the ECG. Using the final probability scores of the ECGs, we searched for an optimal threshold [29] of each class. The optimal thresholds were obtained by applying thresholds between 0 and 1 in increments of 0.01 to achieve the best F1 scores in the validation dataset for each T-NSR and AF-NSR class or T-NSR and CIA-NSR class. The optimal thresholds of each class were saved along with the model weights in the validation phases. The procedures for training and validating with entire 12-lead ECG signals were conducted consistently, using discrete heartbeats as an input, excluding the step of gathering discrete heartbeats.

#### 2.4.4. Ensemble Model for Generalizability

For each architecture, we trained five different models using five different fixed seeds that control random variables for weight initialization, data shuffling, and dropout. Experimenting with five different seeds and ensembling them can be helpful in several ways. First, it can reduce the variance in the model’s performance caused by randomness in the training process. By training the model with different random seeds, we can obtain several different versions of the model, each with its own biases and strengths. Ensembling these models can help to reduce the impact of individual biases and improve the overall performance of the model. Secondly, ensembling models trained with different seeds can provide a more robust estimate of the model’s performance. By combining the outputs of several different models, we can reduce the impact of outliers and obtain a more accurate estimate of the model’s true performance [30].

In the testing phase (Figure 6b), we ensembled the probability value of all five models by averaging the probability values for each class of an ECG. Then, we evaluated those probability values with the averaged thresholds of five models.

#### 2.4.5. Metrics for Model Performance Evaluation

The F1 score, AUC of the ROC (AUROC), precision (positive predictive value), recall, and negative predictive value (NPV) for each T-NSR, AF-NSR pair, and T-NSR, CIA-NSR pair were used to evaluate the performance of our model. The F1 score in Equation (3) is the harmonic mean of the precision (Equation (1)) and recall (Equation (2)). The F1 score is often used as an evaluation metric in various medical AI fields, along with the AUC of the ROC. The AUC of the ROC is a performance metric ranging from 0.5 to 1 that shows the discriminatory ability of the model. The AUC of the ROC alone is not suitable to validate a model’s performance since the AUC of the ROC is sensitive to class-imbalanced datasets, such as our datasets (6.6446 NSR to 1 AF-NSR). In other words, the AUC of the ROC will be biased towards evaluating the majority class: T-NSR ECGs. The NPV in Equation (4) measures the proportion of true negative predictions among all the negative predictions. To carefully evaluate our model to the class-imbalanced dataset, we propose F1, AUROC, precision, recall, and NPV for model evaluation.

Precision = (True Positive)/(True Positive + False Positive) (1)

Recall = (True Positive)/(True Positive + False Negative) (2)

F1 Score = 2/((1/Precision) + (1/Recall)) (3)

NPV = (True Negative)/(True Negative + False Negative) (4)

## 3. Results

### 3.1. Results for Different Architectures

**Figure 7:**
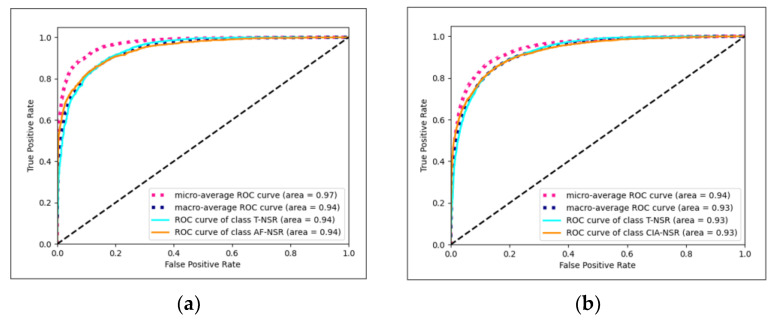
(**a**) Conv1D+LSTM model AUC-ROC plot for T-NSR/AF-NSR prediction; (**b**) ResNet-18 model AUC-ROC plot for T-NSR/CIA-NSR prediction.

The Conv1D+LSTM model exhibited the best performance for T-NSR/AF-NSR, achieving an average AUC of 0.9419, as illustrated in Figure 7a. Meanwhile, the ResNet-18 model stood out for T-NSR/CIA-NSR, with an average AUC of 0.9272, depicted in Figure 7b.

The findings from our study indicate that utilizing discrete heartbeats from normal sinus rhythm ECG signals as the input in deep learning models demonstrated higher efficacy in predicting future occurrences of arrhythmia and atrial fibrillation, as evident from the outcomes presented in Table 3, Table 4, Table 5 and Table 6. Specifically, for the analysis of T-NSR and CIA-NSR in Table 4, the LSTM model trained with discrete heartbeats achieved an AUC score of 0.9222, outperforming the LSTM model trained with entire 12-lead ECG signals, which achieved an AUC score of 0.8909. Similarly, for the analysis of T-NSR and AF-NSR, the LSTM model utilizing discrete heartbeats achieved an average AUC score of 0.9419, surpassing the AUC score of 0.9124 obtained by the LSTM model trained with entire 12-lead ECG signals.

### 3.2. Paired t-Test for Discrete Heartbeat and 12-Lead Input

We sought to statistically compare the performance of two modeling methods using a paired *t*-test. A paired t-test is suitable in this context because it evaluates if there’s a significant difference between two paired groups. The “pairing” in our case came from evaluating the two input methods on the same dataset across five different seed models. The null hypothesis (H0) for our test was set as: “There is no significant difference between the performance metrics of the two input methods”. Conversely, the alternative hypothesis (H1) was set as: “There is a significant difference between the performance metrics of the two input methods.” The metrics of interest in our study were the F1 score and AUROC. The results consistently indicate *p*-values less than the significance level of 0.05 for both the F1 and AUROC across all models, as shown in Table 7, Table 8, Table 9 and Table 10.

A *p*-value below the 0.05 threshold is typically interpreted as strong evidence against the null hypothesis in many scientific disciplines. It suggests that the observed data (in our case, the differences in the performance metrics between the two methods) would be unlikely if the null hypothesis were true. Therefore, we reject the null hypothesis in favor of the alternative hypothesis, suggesting that there was a significant difference between the performance metrics of the two input methods.

## 4. Discussion

This study presents evidence of the effectiveness of using discrete heartbeats extracted from normal sinus rhythm ECGs in predicting future arrhythmia and atrial fibrillation incidences with deep learning methods. The results of the study also suggest that a specific biomarker for future incidences of arrhythmia and atrial fibrillation may be present in the normal sinus rhythm ECG signal.

It is worth noting that the study only utilized signals from ECG recordings and did not incorporate additional patient information, such as electronic medical records, which may raise concerns about privacy and the potential compromise of patient confidentiality. Despite relying on limited data, the study still demonstrates high performance in predicting future arrhythmia and atrial fibrillation incidences from normal sinus rhythm ECGs, suggesting that ECG signals alone may be sufficient for accurate prediction. This finding is promising, as it indicates that analyzing discrete heartbeats extracted from normal sinus rhythm ECGs may facilitate efficient and precise diagnosis and treatment without requiring extensive patient information.

Our dataset observed a pronounced proportion of CIA-NSR to T-NSR, with a ratio of 11,929 to 35,455, equating to approximately 33.6%. This stands in contrast to general population statistics, where the prevalence of arrhythmias is around 5% [31]. People typically seek hospital care for distinct health concerns, especially those related to cardiac issues. Consequently, the dataset may naturally represent a heightened occurrence of CIA, mirroring a patient group more prone to cardiac irregularities. While this offers insight into real-world situations, it may not accurately reflect the distribution in the wider community.

In artificial intelligence research using ECGs, there have been studies that predict the clinical data of patients. Several studies have successfully indicated patients’ clinical data, such as gender classification, age prediction, and heart failure prognosis [32,33,34]. These endeavors have been recognized for their accuracy, highlighting that the clinical information is already present in the ECG signals. Even without the help of artificial intelligence, anatomical and electrophysiological remodeling of the heart is reflected in the ECGs of patients with arrhythmias, including atrial fibrillation [35].

There are several limitations to the study that should be taken into consideration. Firstly, the research relied on data from only two hospitals, an aspect that inherently needs broader external validation. Broadening our data sources to encompass more hospitals or diverse patient groups would enhance the robustness and generalizability of our conclusions. Secondly, it is essential to acknowledge that the T-NSR ECGs examined in this study might inadvertently encompass instances of AF-NSR or CIA-NSR due to the absence of continuous data for labeling. This potential overlap exists despite our rigorous data collection from patients who had three or more T-NSR ECGs within a year and exhibited no clinical symptoms of AF or CIA during medical evaluations by physicians. Such challenges persist in intermittent electrocardiogram research unless continuous monitoring is employed, like long-term implantable loop recorders [36]. To overcome this limitation, we are exploring incorporating data from wearable devices for 24 and 74 h immediately following the 12-lead electrocardiogram recording as a follow-up study. Lastly, our research was retrospective, and it is recognized that a prospective study would offer a more rigorous evaluation of our findings. Recognizing this need, we initiated the “PROVISION-AF trial” in February 2023, a prospective, multicenter study registered with ClinicalTrials.gov under NCT05725187. This forward-looking approach aims to validate and potentially refine our model in real-time scenarios, enhancing its reliability and adaptability across a broader range of healthcare contexts.

For future research, it would be beneficial to investigate the specific heartbeats within individual electrocardiogram signals that predict the future incidence of atrial fibrillation and arrhythmia. By identifying these heartbeats, we can determine which components or features of discrete heartbeats act as potential biomarkers. Additionally, we could group study participants based on relevant demographic and medical factors and assign distinct threshold values to each subgroup to gain more nuanced insights into the predictive value of specific discrete heartbeat features for arrhythmia and atrial fibrillation. These approaches could provide greater insight into the underlying mechanisms and physiological factors contributing to the development of arrhythmia and atrial fibrillation and potentially develop more personalized diagnostics.

**Figure 8:**
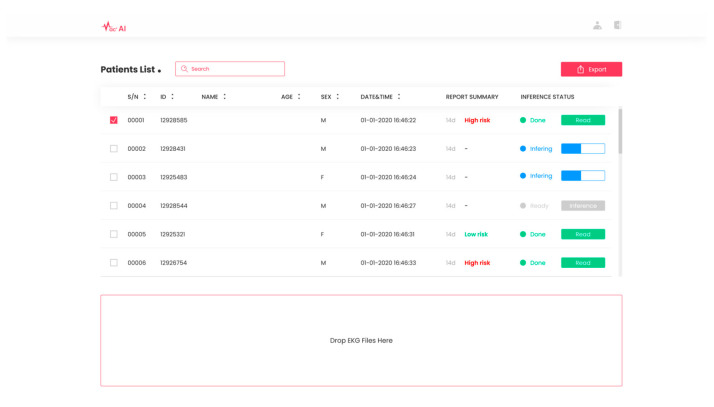
SaaS product SYN-MAC. SaaS product by Synergy A.I. Co., Ltd., located in Seoul, Republic of Korea. for predicting future occurrences of arrhythmias from normal sinus rhythm ECGs.

Based on the study presented in this paper, we obtained approval (Approval No. 2023000086) from the Ministry of Food and Drug Safety in South Korea for our exploratory clinical trials. Leveraging the deep learning-based cardiac arrhythmia prediction, we have developed SYN-MAC, a software-as-a-service (SaaS) product in Figure 8 offered by Synergy A.I. Co., Ltd., Seoul, Republic of Korea. This software is designed to predict future incidences of clinically significant arrhythmias and categorize them as “high risk” or “low risk” based on the threshold value of combined discrete heartbeats. With this software, we will conduct additional confirmatory clinical trials in live environments, focusing on enhancing the prediction accuracy of clinically important arrhythmias and advancing AI-based medical technologies for the early detection of diverse heart diseases.

## 5. Conclusions

This study’s results suggest that using discrete heartbeats extracted from normal sinus rhythm ECG signals to predict future clinically important arrhythmia and atrial fibrillation incidences rather than using entire 12-lead ECG signals with deep learning models is a promising approach. The LSTM models for both atrial fibrillation and clinically important arrhythmia prediction using discrete heartbeat showed strong performance compared to using entire 12-lead ECG signals. The study demonstrated that ECG signals alone were sufficient for accurate prediction, and a potential biomarker may be present in the normal sinus rhythm ECG signal. This suggests that using discrete heartbeats with deep learning models may enable the detection of subtle patterns in ECG signals, which could lead to a more accurate and earlier diagnosis of clinically important arrhythmia and atrial fibrillation.

## Figures and Tables

**Table 1:** Data overview. Training, validation, and test data for T-NSR and AF-NSR.

AF-NSR/T-NSR		Number of Heartbeats	Number of ECGs
T-NSR	Training	3,177,263	21,028
	Validation	1,058,107	6972
	Testing	977,875	6514
AF-NSR	Training	604,700	3225
	Validation	198,672	1073
	Testing	228,708	1385

**Table 2:** Data overview. Training, validation, and testing data for T-NSR and CIA-NSR.

CIA-NSR/T-NSR		Number of Heartbeats	Number of ECGs
T-NSR	Training	3,177,263	21,028
	Validation	1,058,107	6972
	Testing	977,875	6514
CIA-NSR	Training	1,113,089	6855
	Validation	375,700	2329
	Testing	423,581	2543

**Table 3:** Test dataset performance evaluation for AF-NSR and T-NSR.

AF-NSR/T-NSR	Input	ResNet-18	Conv1D+ LSTM	Conv1D+ Transformer
Average F1	Heartbeat	0.8468	0.8499	0.8371
	12-Lead	0.8302	0.8078	0.7837
Average AUC	Heartbeat	0.9392	0.9419	0.9318
	12-Lead	0.9278	0.9124	0.8982
T-NSR F1	Heartbeat	0.9580	0.9596	0.9570
	12-Lead	0.9564	0.9499	0.9440
AF-NSR F1	Heartbeat	0.7357	0.7402	0.7171
	12-Lead	0.7039	0.6656	0.6234
T-NSR Precision	Heartbeat	0.9276	0.9367	0.9408
	12-Lead	0.9302	0.9131	0.9070
AF-NSR Precision	Heartbeat	0.7981	0.8108	0.8232
	12-Lead	0.7947	0.7343	0.6618
T-NSR Recall	Heartbeat	0.9904	0.9837	0.9738
	12-Lead	0.9841	0.9898	0.9841
AF-NSR Recall	Heartbeat	0.6823	0.6809	0.6352
	12-Lead	0.6318	0.6087	0.5892
T-NSR NPV	Heartbeat	0.9105	0.8693	0.8124
	12-Lead	0.8639	0.8875	0.8230
AF-NSR NPV	Heartbeat	0.9458	0.9457	0.9382
	12-Lead	0.9378	0.9336	0.9295

**Table 4:** Test dataset performance evaluation for CIA-NSR and T-NSR.

CIA-NSR/T-NSR	Input	ResNet-18	Conv1D+ LSTM	Conv1D+ Transformer
Average F1	Heartbeat	0.8361	0.8365	0.8392
	12-Lead	0.8317	0.8049	0.7903
Average AUC	Heartbeat	0.9272	0.9222	0.9248
	12-Lead	0.9184	0.8909	0.8789
T-NSR F1	Heartbeat	0.9149	0.9131	0.9161
	12-Lead	0.9130	0.8975	0.8904
CIA-NSR F1	Heartbeat	0.7570	0.7601	0.7623
	12-Lead	0.7505	0.7122	0.6902
T-NSR Precision	Heartbeat	0.8675	0.8753	0.8719
	12-Lead	0.8604	0.8425	0.8407
CIA-NSR Precision	Heartbeat	0.8056	0.7731	0.8070
	12-Lead	0.7941	0.6990	0.6682
T-NSR Recall	Heartbeat	0.9678	0.9539	0.9669
	12-Lead	0.9725	0.9602	0.9464
CIA-NSR Recall	Heartbeat	0.7137	0.7475	0.7324
	12-Lead	0.7114	0.7259	0.7137
T-NSR NPV	Heartbeat	0.8827	0.8468	0.8026
	12-Lead	0.8943	0.8414	0.7975
CIA-NSR NPV	Heartbeat	0.8930	0.9027	0.8927
	12-Lead	0.8917	0.8914	0.8852

**Table 5:** Validation dataset performance evaluation for AF-NSR and T-NSR.

AF-NSR/T-NSR	Input	ResNet-18	Conv1D+ LSTM	Conv1D+ Transformer
Average F1	Heartbeat	0.8480	0.8650	0.8373
	12-Lead	0.8502	0.8118	0.7984
Average AUC	Heartbeat	0.9451	0.9523	0.9134
	12-Lead	0.9314	0.9136	0.8842
T-NSR F1	Heartbeat	0.9661	0.9692	0.9242
	12-Lead	0.9674	0.9596	0.9068
AF-NSR F1	Heartbeat	0.7300	0.7607	0.7503
	12-Lead	0.7330	0.6641	0.6900
T-NSR Precision	Heartbeat	0.9469	0.9547	0.8826
	12-Lead	0.9462	0.9309	0.8621
AF-NSR Precision	Heartbeat	0.7520	0.8044	0.7524
	12-Lead	0.8083	0.6978	0.6771
T-NSR Recall	Heartbeat	0.9861	0.9842	0.9699
	12-Lead	0.9895	0.9901	0.9571
AF-NSR Recall	Heartbeat	0.7092	0.7216	0.7482
	12-Lead	0.6705	0.6334	0.7033
T-NSR NPV	Heartbeat	0.8585	0.8550	0.7928
	12-Lead	0.8885	0.8701	0.8078
AF-NSR NPV	Heartbeat	0.9597	0.9616	0.9084
	12-Lead	0.9550	0.9494	0.8996

**Table 6:** Validation dataset performance evaluation for CIA-NSR and T-NSR.

CIA-NSR/T-NSR	Input	ResNet-18	Conv1D+ LSTM	Conv1D+ Transformer
Average F1	Heartbeat	0.8320	0.8268	0.817
	12-Lead	0.8259	0.8064	0.7984
Average AUC	Heartbeat	0.9196	0.9105	0.9108
	12-Lead	0.9144	0.8847	0.8842
T-NSR F1	Heartbeat	0.9223	0.9202	0.9136
	12-Lead	0.9188	0.9117	0.9068
CIA-NSR F1	Heartbeat	0.7417	0.7334	0.7204
	12-Lead	0.7330	0.7011	0.6900
T-NSR Precision	Heartbeat	0.8827	0.8827	0.8845
	12-Lead	0.8742	0.8631	0.8616
CIA-NSR Precision	Heartbeat	0.7676	0.7572	0.7634
	12-Lead	0.7574	0.6938	0.6771
T-NSR Recall	Heartbeat	0.9656	0.9610	0.9447
	12-Lead	0.9682	0.9662	0.9571
CIA-NSR Recall	Heartbeat	0.7175	0.7110	0.6820
	12-Lead	0.7101	0.7085	0.7033
T-NSR NPV	Heartbeat	0.8566	0.8410	0.8024
	12-Lead	0.8674	0.8424	0.8078
CIA-NSR NPV	Heartbeat	0.9076	0.9054	0.9014
	12-Lead	0.9028	0.9019	0.8996

**Table 7:** *p*-values for test dataset between heartbeat and 12-lead inputs for AF-NSR/T-NSR.

AF-NSR/T-NSR	ResNet-18	Conv1D+ LSTM	Conv1D+ Transformer
*p*-value Avg. F1	0.0119	0.0081	0.0230
*p*-value Avg. AUC	0.0393	0.0042	0.0104

**Table 8:** *p*-values for test dataset between heartbeat and 12-lead inputs for CIA-NSR / T-NSR.

CIA-NSR/T-NSR	ResNet-18	Conv1D+ LSTM	Conv1D+ Transformer
*p*-value Avg. F1	0.0434	0.0092	0.009
*p*-value Avg. AUC	0.0253	0.0132	0.0126

**Table 9:** *p*-values for valid. dataset between heartbeat and 12-lead inputs for AF-NSR / T-NSR.

AF-NSR/T-NSR	ResNet-18	Conv1D+ LSTM	Conv1D+ Transformer
*p*-value Avg. F1	0.0089	0.0083	0.015
*p*-value Avg. AUC	0.0165	0.0048	0.0091

**Table 10:** *p*-values for valid. dataset between heartbeat and 12-lead inputs for CIA-NSR / T-NSR.

CIA-NSR/T-NSR	ResNet-18	Conv1D+ LSTM	Conv1D+ Transformer
*p*-value Avg. F1	0.0233	0.0075	0.0106
*p*-value Avg. AUC	0.0212	0.0094	0.0107

## Data Availability

The clinical data were used under IRB approval for use only in the current study. Hence, the dataset used in this study is not publicly available.
